# Microbiological and behavioral determinants of genital HPV infections among adolescent girls and young women warrant the need for targeted policy interventions to reduce HPV risk

**DOI:** 10.3389/frph.2022.887736

**Published:** 2022-07-28

**Authors:** Harris Onywera, Sikhumbuzo A. Mabunda, Anna-Lise Williamson, Zizipho Z. A. Mbulawa

**Affiliations:** ^1^Institute of Infectious Disease and Molecular Medicine, University of Cape Town, Cape Town, South Africa; ^2^Division of Medical Virology, Department of Pathology, Faculty of Health Sciences, University of Cape Town, Cape Town, South Africa; ^3^Division of Medical Microbiology, Department of Pathology, Faculty of Health Sciences, University of Cape Town, Cape Town, South Africa; ^4^Research, Innovations, and Academics Unit, Tunacare Services Health Providers Limited, Nairobi, Kenya; ^5^Research and Innovation, Mount Kenya University, Thika, Kenya; ^6^SAMRC/UCT Gynaecological Cancer Research Centre, University of Cape Town, Cape Town, South Africa; ^7^School of Population Health, University of New South Wales, Sydney, NSW, Australia; ^8^The George Institute for Global Health, University of New South Wales, Sydney, NSW, Australia; ^9^National Health Laboratory Service, Nelson Mandela Academic Hospital, Mthatha, South Africa; ^10^Department of Laboratory Medicine and Pathology, Walter Sisulu University, Mthatha, South Africa

**Keywords:** HPV, determinants, risk factors, adolescent girls and young women (AGYW), South Africa

## Abstract

**Background:**

Genital human papillomavirus (HPV) is the most common sexually transmitted virus in most populations globally. Adolescent girls and young women (AGYW) remain a key population group at risk for HPV infection. However, the risk factors of HPV infection among AGYW, especially in sub-Saharan Africa, are a subject of little investigation in published literature. Here, we investigated the factors associated with HPV infection among unvaccinated South African AGYW with a high HPV burden (prevalence: 76.1%).

**Methods:**

We retrospectively recruited 213 AGYW learners (aged 15–25 years) from a previous cross-sectional study, the HPV Education Intervention Study, conducted in the Eastern Cape, South Africa. Sexually transmitted infections (STIs), bacterial pathobionts, genital ulcers (due to infectious causes), candidiasis, and bacterial vaginosis (BV) in the self-collected vaginal specimens were determined using the Allplex™ Panel Assays. Statistical analyses were performed using STATA v16.1. Continuous and categorical variables were computed by *t*-test /Wilcoxon rank-sum test and Chi-square/Fisher's exact tests, respectively. Logistic regression was used to determine the univariable predictors of HPV infection.

**Results:**

The overall detection rate of any viral STI, bacterial STI, pathobiont, genital ulcer, candidiasis, and BV among the AGYW was 75.0, 34.4, 90.7, 14.4, 26.9, and 43.6%, respectively. The main factors associated with HPV infection were alcohol consumption (*p* = 0.005), infection with any and multiple *Candida* species (*p* = 0.011 and 0.006, respectively), *Candida albicans* infection (*p* = 0.010), *Ureaplasma urealyticum* pathobiont infection (*p* = 0.044), BV-associated bacteria (specifically *Atopobium vaginae*: *p* = 0.039, BV-associated bacteria 2: *p* = 0.021, *Gardnerella vaginalis*: *p* = 0.021, *Megasphaera* type 1: *p* = 0.037), and BV (*p* = 0.011).

**Conclusions:**

Our study, albeit not necessarily generalizable, found social behavior as well as specific vaginal microbes as correlates of HPV infection among AGYW in South Africa. There is a need to investigate HPV epidemiology in other AGYW populations. The factors associated with genital HPV infection among AGYW burdened with HPV infection necessitate the need to formulate and implement population-specific public health strategies for creating HPV awareness and reducing its risk.

## Introduction

Cervical cancer (CC) is the second most common female cancer in women aged 15–44 years in the world, with its magnitude of difference in incidence by geography often striking (1). Sub-Saharan Africa (SSA) bears the highest burden of CC. Worldwide data has consistently reported that genital HPV infection is the most common sexually transmitted infection (STI) in most populations (1, 2), and that persistence of high-risk HPV (HR-HPV) infection causes anogenital cancers (2)—including CC (3). There is extensive overlap between geographical regions significantly burdened with CC and genital HPV infection. Epidemiological studies have found that HPV infection exhibits non-uniform geographical distribution, with SSA having the highest global prevalence (country prevalence ranges from 8.5 to 74.6% among women with normal cervical cytology) (1, 2, 4). Studies have noted a link between HPV infection and the age of women (1, 2, 4). These studies have observed that, whatever the geographical region and period of investigation, the early peak of HPV infection occurs at adolescent and young adult ages (<25 years). Thereafter, HPV prevalence declines with increasing age as observed globally (1, 2). However, in Africa, a second peak, though less pronounced, occurs in older women (≥45 years) (1, 2).

Globally, adolescent girls and young women (AGYW) remain a key sub-population most vulnerable to STIs, including HIV and HPV (4–13). Of note, a study that collated data from various sources in the U.S. found that 48.1% of the reported STIs occurred among youth (aged 15–24 years), of which at least half (50.5%) had incident HPV infections (14). A cross-sectional, multicentric, nationwide Brazilian survey found that 54.6 and 38.6% of sexually active unvaccinated AGYW had HPV and HR-HPV infections, respectively (8). Among indigenous Panamanian AGYW learners, HPV prevalence was found to be 33.2% (10). It has been noted that, among AGYW, the cumulative prevalence of HPV infection can exceed 80%, and that persistent HPV infection, to a lesser extent, is associated with cervical cytologic abnormalities (11). HPV infections among AGYW in SSA are significantly higher (4–6, 12), including in rural settings (12), than in the general populace of SSA and the rest of the globe (1, 2). South Africa still has scarce data on HPV infection among AGYW. The few published studies have reported that HPV and HR-HPV prevalence among AGYW can be as high as over 70 and 50% (4, 6, 12), respectively. These observations are confirmed and reinforced by the current high prevalence of HPV (76.1%) and HR-HPV (54.5%) AGYW from the Eastern Cape Province of South Africa (15). Published data on South African AGYW have also reported that multiple HPV infection varies by geographical area (5). In these HPV studies on South African cohort of AGYW, the determinants of HPV infection remain inconsistent and largely unexplored.

The determinants of HPV infection among women, including AGYW, appear to be very similar. The early peak in HPV prevalence often coincides with age at first sexual intercourse, which occurs during adolescent and young adult ages for most women. Both adolescent age and sexual debut in adolescence have been linked to prevalent HPV (16) and incident HR-HPV infection (17). Epidemiological studies have convincingly shown that HPV infection is rare before the first coitus (7, 13, 18, 19), and that HPV transmission in virgins is partly because of non-penetrative sexual contact (13) or douching habits (7). The rapid acquisition of HPV among AGYW after first coitus emphasizes the importance of vaccinating young girls and adolescents against HPV prior to early sexual debut. Other common risk factors (such as condomless vaginal sex, douching habits, and STIs) for HPV infection have been acknowledged (5, 12, 13, 16, 17, 19–21). Aside from these factors, vaginal microecology is gaining prominence on its role on HPV infection. In a meta-analysis, *Candida albicans* and bacterial vaginosis (BV, a vaginal syndrome-induced by an overgrowth of anaerobic bacteria) were identified as protective and risk factors for HPV infection, respectively (21). BV-associated bacteria (e.g., *Gardnerella vaginalis*) and vaginal microflora with paucity of keystone *Lactobacillus* spp. (e.g., *Lactobacillus cripatus*) are considered less protective against HPV infection (22–24). However, studies of the association of HPV (including HR-HPV) infection with vaginal microecology, specifically *Candida* spp. (20, 25) and *Lactobacillus* spp. (22, 23, 26), have yielded inconsistent and conflicting results. Hitherto, there are conflicting reports over the relationship of the relative abundance or dominance of *L. iners* with HPV infection; non-significant (24), positive (22, 27), and inverse associations (26) have all been reported. This could be ascribed to inter-study heterogeneity and clonal lineages of *L. iners*. Vaginal microecology and HPV have been associated with cervical intraepithelial neoplasia (21). Thus, more studies are needed to evaluate and understand the relationship between vaginal microecology and HPV infection, if we are to identify modifiable risk factors for HPV and CC prevention.

In Southern Africa, CC remains the most common cancer affecting women aged 15 to 44 years (1). Prophylactic HPV vaccines have been licensed in several countries as a primary preventive strategy for cervical HPV disease. Currently, two HPV vaccines are licensed in South Africa: (i) Cervarix®–a bivalent vaccine targeting two HR-HPV types (HPV16 and HPV18, responsible for majority of cancer, including CC), and (ii) Gardasil®4—a quadrivalent vaccine targeting HPV16, HPV18, and two low-risk HPV types (HPV6 and HPV11, the most common causative agents of genital warts) (28). Gardasil®4, administered in three doses (0, 1–2, 6 month schedule), was successfully used in an HPV vaccination demonstration programme in South Africa targeting girls aged 9–12 years (29). Nevertheless, in April 2014, South Africa's national Department of Health introduced a school-based HPV vaccination programme; with the administration of a two-dose regimen (6 months apart schedule) of the Cervarix®, primarily targeting girls aged ≥9 years in their fourth year of elementary (primary) school (Grade 4, equivalent to 4th Grade in the U.S. and Year 5 in the UK) (30). To realize the greatest impact of the HPV vaccination programme, the World Health Organization (WHO) recommends that the vaccines be administered to young girls (9–13 years) prior to sexual debut, hence before exposure to HPV infection (28). Most AGYW in South Africa do not have access to the HPV vaccine programme since only girls in Grade 4 in some public schools are prioritized (31). For AGYW aged 15–25 years who may not benefit from catch-up vaccination programmes, an understanding of the modifiable risk factors may help to reduce the duration of HPV infection. To address this, information on HPV epidemiology among AGYW need to be collected.

We are currently aware of the high HPV prevalence, including the genotypes targeted by Gardasil®9 (a non-avalent HPV vaccine) among unvaccinated AGYW in rural Eastern Cape (15), yet HPV risk factors among AGYW remain unknown. Thus, the aim of the present study was to determine the factors associated with genital HPV infection among AGYW in the Eastern Cape. This study may help formulate and implement evidence-based policies designed to create and intensify awareness of risk factors for HPV infection among AGYW in SSA.

## Materials and methods

### Study design, setting, and study subjects

This was a retrospective cross-sectional descriptive study among female learners who had participated in a parent study known as the HPV Education Intervention Study (32) that was conducted between April and May 2019. All the study participants were from two high schools in Chris Hani District Municipality, Eastern Cape Province (South Africa). The participating high schools were randomly selected, and they belong to quintile one (no-fee paying schools) South African Department of Education quintile ranking. Participants were invited to and recruited from the nearest primary care facilities. Upon being conversant with the study, participants filled out a detailed questionnaire, mostly with closed-format questions about their demographics, sexual behavior, alcohol consumption, and smoking habits. All information collected from the study participants was anonymised and de-identified prior to analysis. Participants did not include their identities on the questionnaires. To be eligible for participation in the present study, the female had to be aged ≥15 and ≤25 years, and sexually experienced. Any AGYW who was menstruating or pregnant at the time of vaginal specimen collection on the day of the visit was excluded from the study. In the assessment of the factors associated with HPV infection, only AGYW with valid HPV results as performed by the HPV genotyping assay (described in a later subsection) were included.

### Collection of clinical specimens and HIV test

Details regarding specimen collection used for evaluation of STIs, vaginal bacterial pathobionts, genital ulcers, and BV are published elsewhere (15). A pathobiont is a potentially pathological organism which, under normal microenvironment lives as a non-harming symbiont but causes pathogenesis under an altered ecosystem condition. In brief, with regard to sample collection, a health professional trained the AGYW on the self-sampling technique using the Evalyn® Brush (Rovers® Medical Devices B.V., Oss, Netherlands) as shown in the information leaflet. Specimen was collected by inserting the white brush as far as possible into the vagina after assuming a comfortable stance (either while standing or squatting), and then rotating the plunger 360° five times. The brush was carefully removed from the vagina and capped back after being pulled by the pink plunger into the transparent casing. Brushes with self-collected vaginal specimens were stored at room temperature and shipped to the HPV Laboratory at the University of Cape Town, South Africa for laboratory processing.

HIV counseling and testing was performed at the primary care facilities by either qualified clinic staff or HIV lay counselor. HIV test was done using a rapid antibody screening test using blood from a finger prick. All newly diagnosed HIV-infected AGYW were followed-up according to the Department of Health protocol for management of HIV/AIDS in adults.

### Laboratory processing of specimens and molecular detection of STIs, genital ulcers, candidiasis, and BV

Nucleic acid from each specimen was isolated as detailed elsewhere (15) using MagNA Pure Compact (Roche Molecular Systems, Inc., Branchburg, NJ, USA) and MagNA Pure Compact Nucleic Acid Isolation Kit (Roche Molecular Systems, Inc., Branchburg, NJ, USA), according to the manufacturer's instructions.

HPV DNA testing in the specimens was performed using the Roche Linear Array HPV Genotyping Assay (Roche Molecular Systems, Inc., Branchburg, NJ, USA) according to the manufacturers' instructions. The primers for this assay are designed to amplify a 450-bp fragment of the L1 region of 13 high-risk and 24 low-risk HPV genotypes [as stated elsewhere (15)] as well as a 268-bp fragment of the β-globin gene. The β-globin gene was amplified concurrently to monitor and assess cellular adequacy, extraction, and amplification for each processed specimen.

To detect STIs, bacterial pathobionts, genital ulcers, candidiasis, and BV in the vaginal swabs, we used a multiplex real-time PCR assay, the Allplex™ Panel Assays (Seegene Inc., Seoul, Korea), according to the manufacturers' instructions. The Allplex™ STI Essential Assay (Seegene Inc., Seoul, Korea) detects and identifies the following two bacterial STIs (*Chlamydia trachomatis* and *Neisseria gonorrhoeae*), four pathobionts (*Mycoplasma genitalium, Mycoplasma hominis, Ureaplasma parvum*, and *Ureaplasma urealyticum*) and one parasitic pathogen (*Trichomonas vaginalis*). The pathobionts that we assessed are believed to be emerging sexually transmitted pathogens (33, 34).

Genital ulcers due to infectious causes were diagnosed using the Allplex™ Genital Ulcer Assay (Seegene Inc., Seoul, Korea) whose panel consists of the following pathogens: herpes simplex virus type 1 (HSV-1), HSV-2, cytomegalovirus (CMV), varicella-zoster virus (VZV), *C. trachomatis* serovar L. (causes lymphogranuloma venereum, LGV), *Treponema pallidum* (causes syphilis), and *Haemophilus ducreyi* (causes chancroid).

The Allplex™ Candidiasis Assay (Seegene Inc., Seoul, Korea) panel consisted of seven *Candida* spp. These included *Candida albicans, Candida dubliniensis, Candida glabrata, Candida krusei, Candida lusitaniae, Candida parapsilosis*, and *Candida tropicalis*.

The panel for BV diagnosis in the Allplex™ Bacterial Vaginosis Assay (Seegene Inc., Seoul, Korea) is designed to (i) quantitatively detect *Lactobacillus* spp. (*L. crispatus, Lactobacillus gasseri*, and *Lactobacillus jensenii*), *G. vaginalis*, and *Atopobium vaginae*, and (ii) qualitatively detect *Megasphaera* type 1, *Bacteroides fragilis*, BV-associated bacteria 2 (BVAB2), and *Mobiluncus* spp. (*Mobiluncus mulieris* and *Mobiluncus curtisii*).

In each of the above-mentioned four-panel assays, an internal control (an endogenous human gene) was coamplified simultaneously with the target DNA sequence to monitor sample adequacy, nucleic acid extraction, and check for any possible PCR inhibition. RNase-free water and a mixture of pathogen clones were used as negative and positive controls, respectively. Each 20.0 μl reaction consisted of 15.0 μl PCR mastermix and 5.0 μl of template or controls (either negative or positive control). The Allplex™ Panel Assays were all run on a real-time PCR instrument and results were examined using CFX96™ Real-time PCR Detection System (Bio-Rad)—CFX Manager™ Software-IVD v1.6 and CFX96™ Dx System (Bio-Rad)—CFX Manager™ Dx Software v3.1. Seegene Viewer Software (Seegene Inc., Seoul, Korea) was used to interpret data according to the manufacturer's instructions.

### Statistical analysis

Data were captured in Microsoft Excel 2016 (Microsoft Corporation, Seattle, USA) and exported to STATA v16.1 (Stata Corp LP, College Station, Texas, USA) for analysis. Numerical data were explored using the Shapiro Wilk test for normality. Numerical variables that were normally distributed are summarized using the minimum, maximum, mean, and standard deviation. Conversely, numerical variables that were skewed were summarized using the median and interquatile range (IQR). The two-sample *t*-test with equal variances was used to compare the mean age of participants by HPV status, whereas the Wilcoxon rank-sum test was used to compare all other medians of numerical variables by HPV status.

Percentages and frequencies were used to summarize categorical variables. The Chi-squared and Fisher's exact tests were used to compare two categorical variables. For the chi-squared test to be used, the expected frequencies needed to be ≥5, unless if the number of rows being compared exceeded 2 rows, wherein no more than 20% of the rows could have an expected frequency of <5 and no cell in the table could have an expected frequency of <1. Where these conditions were not met, the Fisher's exact test was used.

Logistic regression was used to determine the univariable predictors of HPV infection as well as history of vaginal discharge or itching, and genital ulcers, blisters, or warts. The odds ratio (OR) is the measure of association used and the 95% confidence interval is used to show the precision of estimates. A *p*-value of < 0.05 is used as the level of significance.

## Results

### Baseline characteristics of study subjects

The baseline characteristics of the 220 AGYW that were finally enrolled in this study are summarized in Table 1 using median (IQR) and proportion (%). A majority of the AGYW were aged 17–20 years (78.7%). Only one young woman (0.5%) was aged 25 years. and were in at least Grade 10 (equivalent to 10th Grade or Sophomore Year in the U.S. and Year 11 in the UK: 85.8%). The median age of sexual debut and frequency of vaginal sex in the past 1 month were 16 years and 2, respectively. Approximately 67.8% of the AGYW were currently on contraception, with condom being the most frequently used contraception (37.3%) by the participant and/or sexual partner. About three-quarters (73.1%) of the AGYW had circumcised male sexual partners. Approximately two-thirds (64.5%) and one-fifth (20.6%) of the AGYW had ever experienced vaginal discharge (or itching) and had genital ulceration (including blisters and warts), respectively.

**Table 1:** Baseline characteristics of the 220 sexually experienced study participants.

**Characteristic**	**All participants**
	***N*** = **220**
**Age (years)**	18 (18–20)
15–16	9.1 (20/220)
17–19	65.0 (143/220)
20–25	25.9 (57/220)
**Level of high school education [% (n/N)]**	
Grade 8	6.4 (14/218)
Grade 9	7.8 (17/218)
Grade 10	26.1 (57/218)
Grade 11	30.3 (66/218)
Grade 12	29.4 (64/218)
**Ever smoked cigarette? [% (n/N)]**	
Yes	16.8 (37/220)
No	83.2 (183/220)
**Ever consumed alcohol? [% (n/N)]**	
Yes	81.8 (180/220)
No	18.2 (40/220)
**Sexual debut^∧^**	16 (15–17)
**Number of lifetime sexual partners^∧^**	2 (1–3)
**If alcohol was taken or drugs were used before the last sexual intercourse [% (n/N)]**	
Yes	5.8 (12/206)
No	94.2 (194/206)
**Whether participant and/or partner are currently using any kind of contraception [% (n/N)]**	
Yes	67.8 (139/205)
No	32.2 (66/205)
**Contraception currently used by participant and/or partner [% (n/N)]**	
None	24.6 (49/199)
Birth control pills	6.5 (13/199)
Condoms	28.1 (56/199)
Intrauterine device (Mirena or ParaGard) or implant	1.0 (2/199)
(Implanon or Nexplanon)	
3-month injectable (Depo-Provera)	21.1 (42/199)
2-month injectable (Nur-Isterate)	15.6 (31/199)
Unknown injectable	2.5 (5/199)
Coitus interruptus (withdrawal) or some other method	0.5 (1/199)
**Condom use by partner during last sexual intercourse [% (n/N)]**	
Yes	39.8 (82/206)
No	60.2 (124/206)
**Number of vaginal sex in the past 1 month^∧^**	2 (1–3)
**Experienced vaginal discharge or itching [% (n/N)]**	
Yes	64.5 (142/220)
No	35.5 (78/220)
**Ever had ulcers/blisters/warts on the genitals? [% (n/N)]**	
Yes	20.6 (45/218)
No	79.4 (173/218)

Continuous variables are expressed as medians with interquartile ranges (IQRs = 75th percentile−25th percentile).

∧ Data was not available on the age at sexual debut for one girl, number of lifetime sexual partners for 14 girls, number of lifetime sexual partners for 14 girls, number of vaginal sex in the past 1 month for 11 girls, number of anal sex in the past 1 month for 12 girls, and number of oral sex in the past 1 month for 10 girls.

### Proportion of AGYW positive for STIs, bacterial pathobionts, genital ulcers, candidiasis, and BV

The prevalences of STIs, pathobionts, genital ulcers, candidiasis, and BV among the AGYW are summarized in Table 2. The proportion of any viral STI was high (75.0%). The proportions of these STIs were as follows: HIV (5.3%), HPV (76.1), HSV-1 (0.5%), and HSV-2 (6.0%). The overall prevalence of genital ulcers due to infectious causes (including HSV-1 and HSV-2) was 14.4%, with only one woman (0.5%) having genital ulcers due to multiple infectious causes (CMV and HSV-2 coinfection). CMV was the most common cause of genital ulcers (8.4%). None of the women had genital ulcers associated with VZV, LGV, syphilis, and/or chancroid. Only 26.9% of the AGYW had any detectable fungi (*C. albicans*: 23.6%, *C. glabrata*: 4.8%, and *C. lusitaniae*: 1.4%). Multiple fungal infections were at least 8 times more prevalent than single fungal infections (89.3 vs. 10.7%).

**Table 2:** Proportion of AGYW with STIs, genital ulcers, candidiasis, and BV.

**Molecular diagnosis characteristic**	**All participants**
	***N* = 220**
**Viral STIs^$^ [% (n/N)]**	
Any viral STI	75.0 (165/220)
Single viral STI	88.5 (146/165)
Multiple viral STI	11.5 (19/165)
Any HPV type	76.1 (162/213)
Any high-risk HPV type	54.5 (116/213)
Single HPV infection	17.8 (38/213)
Multiple HPV infection	58.2 (124/213)
HIV infection	5.3 (8/152)
HSV-1 infection	0.5 (1/215)
HSV-2 infection	6.0 (13/2015)
**Genital ulcers due to infectious causes^¶^[% (n/N)]**	
Genital ulcers due to any infectious cause	14.4 (31/215)
Genital ulcers due to single infectious cause	14.0 (30/215)
Genital ulcers due to multiple infectious causes	0.5 (1/215)
**CMV infection [% (n/N)]**	
Negative	91.6 (197/215)
Positive	8.4 (18/215)
**Fungal infection (candidiasis) [% (n/N)]**	
Any *Candida* species^*^	26.9 (56/208)
*Candida albicans*	23.6 (49/208)
*Candida glabrata*	4.8 (10/208)
*Candida lusitaniae*	1.4 (3/208)
Single infections	10.7 (6/56)
Multiple infections	89.3 (50/56)
**Bacterial STIs and pathobionts^#^ [% (n/N)]**	
Any bacterial STI and/or pathobiont	91.6 (197/215)
Any bacterial STI	34.4 (74/215)
Any bacterial pathobiont	90.7 (195/215)
Ureaplasma urealyticum^*^	43.7 (94/215)
Ureaplasma parvum^*^	66.8 (148/215)
Mycoplasma hominis^*^	55.8 (120/215)
Mycoplasma genitalium^*^	6.5 (14/215)
*Neisseria gonorrhoeae*	12.1 (26/215)
*Chlamydia trachomatis*	29.8 (64/215)
Single bacterial STI and/or pathobiont infection	25.4 (50/197)
Multiple bacterial STI and/or pathobiont infection	74.6 (147/197)
Single bacterial STI	78.4 (58/74)
Multiple bacterial STI	21.6 (16/74)
Single bacterial pathobiont infection	33.8 (66/195)
Multiple bacterial pathobiont infections	66.2 (129/195)
**Protozoal STI (*Trichomonas vaginalis*) [% (n/N)]**	
Negative	90.8 (197/217)
Positive	9.2 (20/217)
**Bacterial vaginosis^&^ [% (n/N)]**	
Negative^¥^	29.8 (65/218)
Negative^§^	14.2 (31/218)
Intermediate	12.4 (27/218)
Positive	43.6 (95/218)

STI, sexually transmitted infections; HIV, human immunodeficiency virus; HPV, human papillomavirus; HSV-1, herpes simplex virus type 1; HSV-2, herpes simplex virus 2 type 2; CMV, cytomegalovirus.

¶ infectious causes of genital ulcers included HSV-1/2, syphilis (T. pallidum), chancroid (Haemophilus ducreyi), varicella-zoster virus, CMV, granuloma inguinale (donovanosis), lymphogranuloma venerum (Chlamydia trachomatis serotypes L1, L2, L3).

# Bacterial STIs that were detected included Ureaplasma urealyticum, Ureaplasma parvum, Mycoplasma hominis, Mycoplasma genitalium, Neisseria gonorrhoeae, and Chlamydia trachomatis.

* Pathobionts: bacteria that may become pathogenic.

& Bacterial vaginosis was identified by quantitative detection of Lactobacillus spp., Gardnerella vaginalis, Atopobium vaginae, Bacteroides fragilis, Mobiluncus spp., Megasphaera type 1, and BV-associated bacteria 2 (BVAB2) using a multiplex real-time PCR assay.

¥ Normal vaginal microflora, with appreciable numbers of Lactobacillus spp. Quantitative thresholds (log) for Lactobacillus spp. were all between 3.74 and 7.14, as autodetected by the Allplex™ Bacterial Vaginosis Assay.

§ Normal vaginal microflora but lacking appreciable numbers of Lactobacillus spp. Quantitative thresholds (log) for Lactobacillus spp. were between 0.78 and 3.37, as autodetected by the Allplex™ Bacterial Vaginosis Assay.

**Figure 1:**
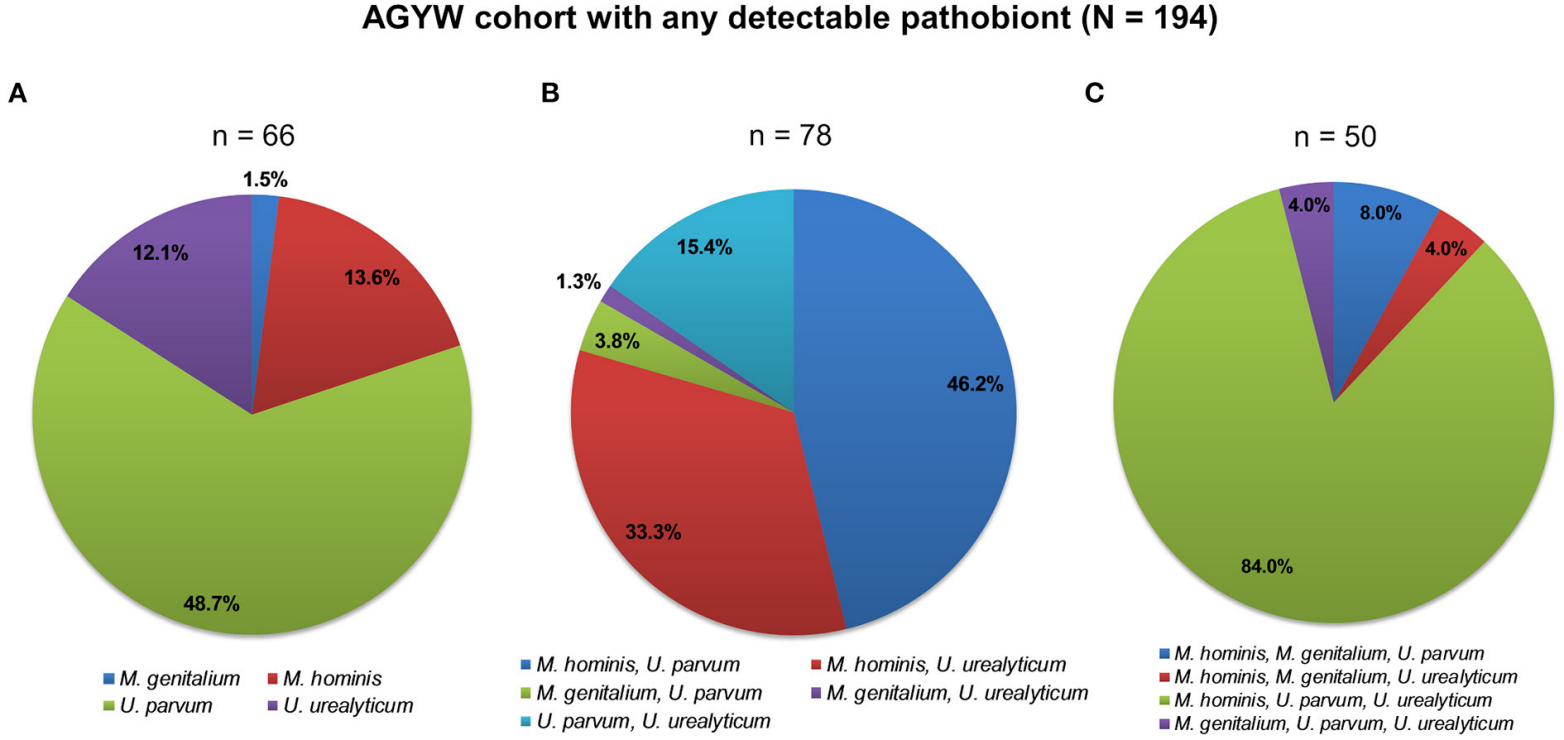
Patterns of bacterial pathobiont infections among 194 AGYW. AGYW with **(A)** single infections, **(B)** dual infections, and **(C)** triple infections. The number of AGYW in each group is in parentheses. In total, 195 AGYW had any detectable pathobiont (*M. genitalium, M. hominis, U. parvum*, or *U. urealyticum*). One participant, not included in any of the two-dimensional pie charts showing the patterns of infections, had all the four examined pathobionts detected.

The overall detection rate of any bacterial STI among the AGYW was 34.4% (*C. trachomatis*: 29.8% and *N. gonorrhoeae*: 12.1%). Single bacterial STI (infection with either *C. trachomatis* or *N. gonorrhoeae*) was more common compared to coinfections (78.4 vs. 21.6%). Most of the AGYW had any pathobiont (overall prevalence: 90.7%—*U. parvum*: 66.8%, *M. hominis*: 55.8%, *U. urealyticum*: 43.7%, and *M. genitalium*: 6.5%), with 66.2% of them having multiple infections. Figure 1 shows the distributions of any detectable pathobiont according to multiplicity of infection (whether single or multiple infections) among AGYW.

Among the single pathobiont infections, *U. parvum* was the most detected (48.7%). Coinfection with *M. hominis* and *U. parvum* was most frequent among dual infections. Simultaneous infection with *M. hominis, U. parvum*, and *U. urealyticum* was the commonest infection with three pathobionts. Only one participant had infections with all the pathobionts. Analogous patterns of infections were observed according to the HPV status of the AGYW (Supplementary Figure 1).

Only 9.2% of the AGYW had protozoal STI (*T. vaginalis*). Lastly, 44.0% of the AGYW had normal vaginal microflora. Of these, 67.7% had vaginal microflora with appreciable numbers of *Lactobacillus* spp. Among AGYW with abnormal microflora, 43.6 and 12.4% had BV and intermediate microflora, respectively.

### Study participant characteristics at study baseline according to HPV status

Of the 220 AGYW, only 213 (96.7%) had valid HPV results as performed by the HPV genotyping assay. A statistical comparison of the baseline characteristics of the 213 AGYW with and without HPV infection is summarized in Table 3. There were statistically significant differences with regard to alcohol consumption (*p* = 0.004), number of lifetime sexual partners (*p* = 0.049), frequency of vaginal sex in the past 1 month (*p* = 0.005), and detectability of the following: any *Candida* spp. (*p* = 0.007), *C. albicans* (*p* = 0.007), multiplicity of *Candida* infection (*p* = 0.013), any bacterial STI/pathobiont (*p* = 0.007), any pathobiont (*p* = 0.004; specifically *U. urealyticum*: *p* = 0.042), BV-associated bacteria (specifically *A. vaginae*: *p* = 0.037, BVAB2: *p* = 0.019, *G. vaginalis*: *p* = 0.020, *Megasphaera* type 1: *p* = 0.034), and BV (*p* = 0.032).

**Table 3:** Comparison of baseline characteristics between HPV-negative and HPV-positive AGYW in South Africa.

**Characteristic**	**HPV-positive** ***N*** = **162**		**HPV-negative** ***N*** = **51**		***p*** **-value**
**Age (years); mean ±sd (min–max)**	18.7 ± 1.6	(15–25)	18.5 ± 1.7	(15–23)	0.542^π^
**Age (years); *n* (%)**					
15–16	14	(73.7)	5	(26.3)	0.953^§^
17–19	105	(76.6)	32	(23.4)	
20–25	43	(75.4)	14	(24.6)	
**Ever consumed alcohol?; *n* (%)**					
Yes	140	(80.0)	35	(20.0)	0.004^§^
No	22	(57.9)	16	(42.1)	
**Sexual debut age (years)^**#**^; median (IQR)**	16.0	(15–17)	15.0	(15–17)	0.293^¥^
**Number of lifetime sexual partners^**~**^; median (IQR)**	2	(2–3)	2	(1–2)	0.049^¥^
**Currently on contraceptive; *n* (%)**					
Yes	107	(79.9)	27	(20.2)	0.979^§^
No	51	(79.7)	13	(20.3)	
**Condom use during last intercourse^**~**^; *n* (%)**					
Yes	60	(79.0)	16	(21.1)	0.902^§^
No	98	(79.7)	25	(20.3)	
**Number of vaginal sex in the past month^**♣**^; median (IQR)**	2	(1–3)	1	(0–2)	0.005^¥^
**Vaginal sex in the past month^**♣**^; *n* (%)**					
None	30	(18.9)	14	(32.6)	0.083^§^
1–2	76	(47.8)	22	(51.2)	
3–4	38	(23.9)	6	(14.0)	
≥5	15	(9.4)	1	(2.3)	
**Experienced vaginal discharge or itching; *n* (%)**					
Yes	108	(78.3)	30	(21.7)	0.306^§^
No	54	(72.0)	21	(28.0)	
**Vaginal discharge or itching last experienced^**α**^; *n* (%)**					
0–7 days	32	(78.1)	9	(22.0)	0.476^§^
8–30 days	17	(68.0)	8	(32.0)	
1–6 months	24	(80.0)	6	(20.0)	
≥6 months	37	(84.1)	7	(15.9)	
**Last experience of genital ulcers/blisters/warts^**Ω**^; *n* (%)**					
0–7 days	13	(61.9)	8	(38.1)	0.657^*^
7–30 days	7	(87.5)	1	(12.5)	
1–6 months	3	(75.0)	1	(25.0)	
≥6 months	7	(77.8)	2	(22.2)	
**Viral STIs; *n* (%)**					
HIV infection	6	(75.0)	2	(25.0)	1.000^*^
HSV-1 infection	1	(100.0)	0	(0.0)	1.000^*^
HSV-2 infection	12	(92.3)	1	(7.7)	0.198^*^
Any viral STI	19	(86.4)	3	(13.6)	0.232^§^
**Genital ulcers due to infectious causes; *n* (%)**					
Positive	26	(83.9)	5	(16.1)	0.270^§^
Negative	136	(74.7)	46	(25.3)	
**CMV infection; *n* (%)**					
Positive	13	(72.2)	5	(27.8)	0.690^§^
Negative	149	(76.4)	46	(23.6)	
**Candidiasis; *n* (%)**					
Any *Candida* species	50	(89.3)	6	(10.7)	0.007^§^
*Candida albicans*	44	(89.8)	5	(10.2)	0.007^§^
*Candida glabrata*	8	(80.0)	2	(20.0)	1.000^§^
*Candida lusitaniae*	1	(33.3)	2	(66.7)	0.152^§^
Single infections	47	(94.0)	3	(6.0)	0.013^*^
Multiple infections	3	(50.0)	3	(50.0)	
**Bacterial STIs and pathobionts; *n* (%)**					
Any bacterial STI and/or pathobiont	153	(78.5)	42	(21.5)	0.007^§^
Any bacterial STI	59	(80.8)	14	(19.2)	0.239^§^
Any bacterial pathobiont	152	(78.8)	41	(21.2)	0.004^§^
*Ureaplasma urealyticum*	77	(82.8)	16	(17.2)	0.042^§^
*Ureaplasma parvum*	117	(79.1)	31	(21.0)	0.122^§^
*Mycoplasma hominis*	94	(80.0)	24	(20.3)	0.169^§^
*Mycoplasma genitalium*	13	(92.9)	1	(7.1)	0.196^*^
*Neisseria gonorrhea*	20	(76.9)	6	(23.1)	0.912^§^
*Chlamydia trachomatis*	51	(81.0)	12	(19.1)	0.278^§^
^∧^ Single bacterial STI and/or pathobiont infection	34	(73.9)	12	(26.1)	0.391^§^
^∧^ Multiple bacterial STI and/or pathobiont infection	119	(79.9)	30	(20.1)	
^&^ Single bacterial STI	47	(82.5)	10	(17.5)	0.503^§^
^&^ Multiple bacterial STI	12	(75.0)	4	(25.0)	
^¶^ Single bacterial pathobiont infection	47	(72.3)	18	(27.7)	0.119^§^
^¶^ Multiple bacterial pathobiont infection	105	(82.0)	23	(18.0)	
***Trichomonas vaginalis*; *n* (%)**					
Positive	13	(65.0)	7	(35.0)	0.224^§^
Negative	149	(77.2)	44	(22.8)	
**Selected vaginal bacteria; *n* (%)**					
*Lactobacillus* species^z^	76	(71.7)	30	(28.3)	0.138^§^
*Atopobium vaginae*	120	(80.0)	30	(20.0)	0.037^§^
BV-associated bacteria 2	71	(84.5)	13	(15.5)	0.019^§^
*Bacteroides fragilis*	13	(81.3)	3	(18.8)	0.767^*^
*Gardnerella vaginalis*	151	(78.2)	42	(21.8)	0.020^§^
*Megasphaera* type 1	61	(84.7)	11	(15.3)	0.034^§^
*Mobiluncus* species	91	(79.1)	24	(20.9)	0.255^§^
**Bacterial vaginosis; *n* (%)**					
Negative	68	(73.9)	24	(26.1)	0.032^§^
Intermediate	16	(59.3)	11	(40.7)	
Positive	78	(83.0)	16	(17.0)	

sd, standard deviation; IQR, interquartile range; STI, sexually transmitted infections.

# N = 212;

♣ N = 202;

~ N = 199;

∧ N = 195;

¶ N = 193;

α N = 140;

& N = 73;

Ω N = 42.

z Lactobacillus species examined included L. crispatus, L. gasseri, and L. jensenii.

π The two sample t-test was used.

§ The Chi-squared statistic was used.

¥ The Wilcoxon rank-sum test was used.

* The Fisher's exact test was used.

For ease of quick inspection, statistically significant results have their p-values indicated in bold.

Overall, HPV-positive AGYW had significantly higher prevalence or frequency of the aforesaid variables compared to HPV-negative AGYW. None of the 213 AGYW with and without HPV infection were coinfected with HIV and HSV-1/2.

### Factors associated with HPV infection, vaginal discharge or itching, and genital ulcers, blisters, or warts

Next, we used logistic regression to determine the factors associated with HPV infection, vaginal discharge or itching, and genital ulcers, blisters, or warts. Table 4 shows that the main factors that were associated with HPV infection comprised alcohol consumption (*p* = 0.005), infection with any *Candida* spp. (*p* = 0.011), *C. albicans* infection (*p* = 0.010), multiple *Candida* infection (*p* = 0.006), infection with any bacterial STI/pathobiont (*p* = 0.010), detectability of any pathobiont (*p* = 0.004—mostly *U. urealyticum*: *p* = 0.044), BV-associated bacteria (specifically *A. vaginae*: *p* = 0.039, BVAB2: *p* = 0.021, *G. vaginalis*: *p* = 0.021, *Megasphaera* type 1: *p* = 0.037), and BV (*p* = 0.011). It is worthwhile to mention that, although not statistically significant, alcohol consumption (*p* = 0.057), consumption of different alcoholic drinks (*p* = 0.0712), and vaginal sex in the past month (*p* = 0.057) were more common in HPV-positive AGYW than HPV-negative AGYW.

**Table 4:** Univariate analysis of predictors of HPV status among AGYW in South Africa.

**Variable**	**% (n/N)**	**OR**	**95% CI**	***p*** **-value**
**Age (years)**				
15–16	73.7 (14/19)	Ref	–	–
17–19	76.6 (105/137)	1.2	(0.4–3.5)	0.777
20–25	75.4 (43/57)	1.1	(0.5–1.9)	0.858
**Vaginal sex in the past month**				
No	68.2 (30/44)	Ref	–	–
Yes	81.6 (129/158)	2.1	(0.1–4.4)	0.057
**Alcohol consumption**				
No	57.9 (22/38)	Ref	–	–
Yes	80.0 (140/175)	2.9	(1.4–6.1)	0.005
**Any *Candida* species**				
No	71.3 (112/157)	Ref	–	–
Yes	89.3 (50/56)	3.3	(1.3–8.4)	0.010
*Candida albicans*				
No	70.7 (111/157)	Ref	–	–
Yes	89.8 (44/49)	3.6	(1.4–9.8)	0.010
**Single *Candida* infection**				
No	50.0 (3/6)	Ref	–	–
Yes	94.0 (47/50)	15.7	(2.2–113.5)	0.006
**Any bacterial STI and/or pathobiont**				
No	50.0 (9/18)	Ref	–	–
Yes	78.5 (153/195)	3.6	(1.4–9.8)	0.010
**Any bacterial STI**				
No	79.2 (103/130)	Ref	–	–
Yes	80.2 (59/73)	1.5	(0.8–3.0)	0.239
**Any bacterial pathobiont**				
No	50.0 (10/20)	Ref	–	–
Yes	78.8 (152/193)	3.7	(1.4–9.5)	0.004
*Ureaplasma urealyticum*				
No	70.8 (85/120)	Ref	–	–
Yes	82.8 (77/93)	2.0	(1.0–3.9)^¥^	0.044
*Atopobium vaginae*				
No	66.7 (42/63)	Ref	–	–
Yes	80.0 (120/150)	2.0	(1.0–3.9)^¥^	0.039
**BV Associated bacteria 2**				
No	70.5 91/129	Ref	–	–
Yes	84.5 (71/84)	2.3	(1.1–4.6)	0.021
*Gardnerella vaginalis*				
No	55.0 (11/20)	Ref	–	–
Yes	78.2 (151/193)	2.9	(1.1–7.6)	0.025
***Megasphaera* type 1**				
No	71.6 (101/141)	Ref	–	–
Yes	84.7 (61/72)	2.2	(1.0–4.6)^¥^	0.037
**Any viral STI**				
No	74.9 (143/191)	Ref	–	–
Yes	86.4 (19/22)	2.1	(0.6–7.5)	0.241
**Bacterial vaginosis**				
Intermediate	59.3 (16/27)	Ref	–	–
Negative	73.9 (68/92)	1.9	(0.8–4.8)	0.145
Positive	83.0 (78/94)	3.4	(1.6–8.6)	0.011

Logistic regression analyses were used.

OR, odds ratio; CI, confidence interval; Ref, reference; STI, sexually transmitted infection.

¥ Confidence interval touches the null due rounding off the values to one decimal place. All these values are >1.

For ease of quick inspection, statistically significant results have their p-values indicated in bold.

Finally, we investigated whether bacterial STI, candidiasis, BV and genital ulcer panel positivity were associated with vaginal discharge or itching, and genital ulcers, blisters, or warts. These results are tabulated in Table 5. Self-reported history of vaginal discharge or itching was significantly associated with alcohol consumption (*p* = 0.005), infection with any *Candida* spp. (*p* = 0.031), *C. albicans* infection (*p* = 0.014), and *A. vaginae* (*p* = 0.006). None of the analyzed variables was a predictor of self-reported genital ulcers, blisters, or warts.

**Table 5:** Factors associated with self-reported vaginal discharge or itching, and genital ulcers, blisters, or warts.

**Variable**	**Experienced vaginal discharge or itching**			**Ever had genital ulcers, blisters, or warts?**		
	**% (n/N)**	**OR (95% CI)**	***p*** **-value**	**% (n/N)**	**OR (95% CI)**	***p*** **-value**
**Age (years)**						
15–16	57.9 (11/19)	Ref	–	36.8 (7/19)	Ref	–
17–19	60.6 (83/137)	1.1 (0.4–3.0)	0.822	17.6 (24/136)	0.4 (0.1–1.0)	0.542
20–25	77.2 (44/57)	2.5 (0.8–7.4)	0.109	21.4 (12/56)	0.5 (0.2–1.4)	0.187
**Vaginal sex in the past month**						
No	63.6 (28/44)	Ref	–	20.5 (9/44)	Ref	–
Yes	67.1 (106/158)	1.2 (0.6–2.3)	0.668	21.8 (34/156)	1.1 (0.5–2.5)	0.848
**Alcohol consumption**						
No	44.7 (17/38)	Ref	–	21.6 (8/37)	Ref	–
Yes	69.1 (121/175)	2.8 (1.4–5.7)	0.005	20.1 (35/174)	0.9 (0.4–2.2)	0.836
**Any *Candida* species**						
No	60.5 (95/157)	Ref	–	20.6 (32/155)	Ref	–
Yes	76.8 (43/56)	2.2 (1.1–4.3)	0.031	19.6 (11/56)	0.9 (0.4–2.0)	0.873
*Candida albicans*						
No	59.9 (94/157)	Ref	–	20.0 (31/155)	Ref	–
Yes	79.6 (39/49)	2.6 (1.2–5.6)	0.014	22.4 (11/49)	1.2 (0.5–2.5)	0.712
**Single *Candida* infection**						
No	83.3 (5/6)	Ref	–	16.7 (1/6)	Ref	–
Yes	76.0 (38/50)	0.6 (0.1–6.0)	0.690	20.0 (10/50)	1.3 (0.1–11.9)	0.846
**Any bacterial STI and/or pathobiont**						
No	72.2 (13/18)	Ref	–	27.8 (5/18)	Ref	–
Yes	64.1 (125/195)	0.7 (0.2–2.0)	0.492	19.7 (38/193)	0.6 (0.2–1.9)	0.418
**Any bacterial STI**						
No	65.7 (92/140)	Ref	–	18.0 (25/139)	Ref	–
Yes	63.0 (46/73)	0.9 (0.5–1.6)	0.695	25.0 (18/72)	1.5 (0.8–3.0)	0.232
**Any bacterial pathobiont**						
No	70.0 (14/20)	Ref	–	30.0 (6/20)	Ref	–
Yes	64.2 (124/193)	0.8 (0.3–2.1)	0.609	19.4 (37/191)	0.6 (0.2–1.6)	0.267
*Ureaplasma urealyticum*						
No	63.3 (76/120)	Ref	–	17.4 (16/92)	Ref	–
Yes	66.7 (62/93)	1.2 (0.7–2.0)	0.614	22.7 (27/119)	0.7 (0.4–1.4)	0.345
*Atopobium vaginae*						
No	50.8 (32/63)	Ref	–	24.2 (15/62)	Ref	–
Yes	70.7 (106/150)	0.2 (1.3–4.3)	0.006	18.8 (28/149)	0.7 (0.4–1.5)	0.376
**BV Associated bacteria 2**						
No	62.8 (81/129)	Ref	–	17.1 (22/129)	Ref	–
Yes	67.9 (57/84)	1.3 (0.7–2.2)	0.450	25.6 (21/82)	1.7 (0.9–3.3)	0.135
*Gardnerella vaginalis*						
No	65.0 (13/20)	Ref	–	25.0 (5/20)	Ref	–
Yes	64.8 (125/193)	1.0 (0.4–2.6)	0.983	19.9 (38/191)	0.7 (0.3–2.2)	0.591
***Megasphaera* type 1**						
No	64.5 (91/141)	Ref	–	19.1 (27/141)	Ref	–
Yes	65.3 (47/72)	1.0 (0.6–1.9)	0.915	22.9 (16/70)	1.3 (0.6–2.5)	0.529
**Bacterial vaginosis**						
Intermediate	66.7 (18/27)	Ref	–	14.8 (4/27)	Ref	–
Negative	57.6 (53/92)	0.7 (0.3–1.7)	0.400	17.4 (16/92)	1.2 (0.4–4.0)	0.753
Positive	71.3 (67/94)	1.2 (0.5–3.1)	0.645	25.0 (23/92)	1.9 (0.6–6.1)	0.272

Logistic regression analyses were used.

OR, odds ratio; CI, confidence interval; Ref, reference; STI, sexually transmitted infection.

For ease of quick inspection, statistically significant results have their p-values indicated in bold.

## Discussion

STIs, including HPV infection, in AGYW is a pressing global health problem (6, 9, 10); resulting in significantly reduced quality of life of infected individuals. In South Africa, there is little to no available regional and nationwide data on the factors associated with HPV infection among AGYW. Therefore, in the present cross-sectional study, we assessed sociodemographic, behavioral, clinical, and microbiological factors associated with the highly prevalent (76%) HPV infection among AGYW in the rural Eastern Cape.

We identified a wide variety of STIs and pathobionts. The prevalence of any bacterial STI (34%), *N. gonorrhea* (7%), and *C. trachomatis* (30%) were intermediate to those reported among South African AGYW from Cape Town (60, 14, and 43%, respectively) and Johannesburg (19, 5, and 17%, respectively) (35). Our data add to the consensus that, after HPV infection, *C. trachomatis* is the second most prevalent STI among AGYW in South Africa (5, 6, 9, 35) and other regions (10, 14). Syphilis, chancroid, and LGV, were not detected whereas *T. vaginalis, M. genitalium*, HIV, HSV-1, HSV-2, and candidiasis were detected at low rates (1–10%), comparable to AGYW cohorts from different regions in South Africa (5, 9, 35). Overall, the distribution pattern of viral and bacterial STIs and pathobionts among the AGYW resembles that previously reported among Eastern Cape women aged ≥30 years attending community-based clinics or CC screenings (36). However, the prevalences of these microbes, including infections, are higher among AGYW compared to the aforesaid Eastern Cape women (37); thus, reflecting high-risk sexual behavior among AGYW. We also affirmed that BV prevalence (44%) is high among South African AGYW, and regionally ranges from 36 to 51% (35). We find the presence of a wide range of STIs among the HPV-burdened unvaccinated AGYW learners worrying, as it may compromise HPV prevention and control. Studies have already found a positive relationship of HPV with other STIs (20, 21, 36). A small cohort study on U.S. adolescents noted that HPV infections were acquired earlier than other STIs (18). This probably corresponds to the early peak of HPV infection. If indeed HPV infections among AGYW precede most of the other STIs, then it makes sense to ensure that, as many young girls as possible are vaccinated against HPV and educated about human reproductive health prior to their sexual debuts. Perhaps these may substantially reduce the burden of HPV and other STIs.

Owing to the high prevalence of HPV infection in our study cohort (15), current and projected (2030) high caseload of CC in Southern Africa (1, 2), variations of HPV risk factors by community (geography) (5, 8, 12, 16), and recent reporting of HPV-related cervical cytologic abnormalities among women aged ≥18 years in rural Eastern Cape (38), we sought to evaluate the determinants of HPV infection among these AGYW. In univariable analysis, alcohol consumption, candidiasis, *U. urealyticum*, BV and specific BV-associated bacteria were strong predictors of HPV infection. We also found that besides alcohol consumption, vaginal discharge or itching was mainly associated with specific vaginal microbes, all of which were associated with HPV infection. A previous study on Swedish cohort aged 17–50 years noted that vaginal discharge and itching were more common in women with vulvovaginal HPV infection (39). However, our study found no direct association between vaginal HPV infection and vaginal discharge or itching. Abnormal vaginal discharge and itching are probably because of vaginal infections or syndromes, including BV, an independent predictor of HPV.

Alcohol consumption is a common behavior associated with HPV infection (16). It may lead to irrational choices, including high-risk sexual activities (e.g., engaging in condomless vaginal sex) that increase the risk for STIs (40). A survey that estimated alcohol-related health problems among U.S. college students (aged 18–24 years) had 8.4% of the respondents attributing the cause of engaging condomless vaginal sex to alcohol consumption (41). However, in our study, condom use was not a determinant of HPV infection, yet there was a trend toward association of both the frequency of alcohol consumption and consumption of different alcoholic drinks in the past month with HPV infection. This could have been influenced by biased self-reporting of condom use, particularly on events that occurred under the influence of alcohol. We know that alcohol consumption may cause memory impairment and blackouts (42). In congruent with other public health experts (43, 44), our findings suggest that didactic public health campaigns advocating for the reduction of high-risk sexual behavior among AGYW are warranted to curtail HPV infection. School-based interventions with risk-modification counseling can control HPV infection, for example, by improving HPV knowledge and awareness (32), intention to use condoms, and uptake of HPV vaccine among adolescents (45). Currently, there is noticeable poor knowledge of HPV, its associated risk factors, and prevention among AGYW (43, 44), including in our study population (32) and other parts of SSA (31).

There is still a gap between the relationship between vaginal microecology (fungi and bacteria in our context) and HPV infection. There have been mixed findings with regards to the association of candidiasis with HPV and HR-HPV infections (20, 21, 25). We found that, in contrast to a previous report (21), *C. albicans* significantly predicted HPV infection. The observed differences probably reflect methodological and true population differences, which include variations in host epithelial cells (46) and failure to resolve dimorphic switches of *C. albicans* (47). Under normal scenario, *Candida* spp. are human commensal microflora that live in harmony with normal microflora. We think that their ability to form biofilm matrix partially account for their inverse association with HPV infection. This is because the dispersed form of *C. albicans* is more virulent than mature *C. albicans* biofilms (47). *C. albicans* positive association with HPV is, perhaps contributed by adhesion and cellular damage to mucosal epithelia (46, 47). A breeched epithelial barrier may then act as a gateway for HPV and replication in the basal layer (48).

The associations of specific BV-associated bacteria, BV, and pathobionts with HPV infection are in common with other reports (21, 23). Serving as an example, Mbulawa et al. (5) reported an association between BV and HPV among AGYW from Cape Town and Johannesburg in South Africa. These findings are not surprising as a majority of women from SSA, including AGYW from South Africa, have cervicovaginal microflora devoid of appreciable quantities of the protective lactobacilli (35, 49). Furthermore, vaginal microflora with higher abundances of keystone lactobacilli are thought to be protective against HPV (HR-HPV) infection whereas those with BV-associated bacteria (including *A. vaginae*) or BV are not (10, 22). In the only study to date of cervicovaginal microflora and HPV infection in South Africa, higher relative abundances of *A. vaginae* and *G. vaginalis* [believed to initiate BV development (50)] were correlated with HPV infection (including HR-HPV) (49). *A. vaginae* and sialidase gene from *G. vaginalis* have been associated with persistent HPV infection (27). The mechanisms that BV-associated bacteria use to contribute to HPV infection partly parallels that of *C. albicans*. An *in vitro* model found that besides BV-associated *G. vaginalis* manifesting propensity to displace protective lactobacilli from HeLa epithelial cells, caused cytopathogenic changes on these epithelial cells (50). Cervicovaginal microflora with *G. vaginalis* dominance has been associated with host epithelial barrier disruption and impaired wound healing (51). As earlier explained, HPV infection of the basal layer requires disruption of epithelial barriers.

With regards to pathobionts, *U. urealyticum*, has been associated with both increased HPV infection (21) and young women age (≤25 years) (52). It is possible that *U. urealyticum* is also co-acquired during the early peak of HPV infection. Also, this assumption is reinforced by the finding that *U. urealyticum* is more frequent in women with BV than healthy women (53) and the possibility of heterosexual exchange of BV-associated bacteria (54). *Ureaplasma*/*Mycoplasma* infections may be risk factors for persistent HR-HPV infection and could be associated with the development of precancerous cervical lesions (55). *U. urealyticum* could be causing pathogenicity by synergistic effects with other pathobionts and BV associated bacteria. This theory stems from the association of *U. urealyticum*/*U. parvum* coinfection with incident *C. trachomatis* (52) and the perspective that *U. urealyticum* might have better survival in symbiotic relationships with BV-associated bacteria (53). In-depth studies are required to delineate the role of *U. urealyticum* and other pathobionts in HPV infection. Since these pathogens are ubiquitously present in the urogenital tract in both healthy and diseased individuals (52, 53), their quantitative and not only qualitative molecular detection should be considered in order to improve their predictive values (56). Limited knowledge of the relationship between vaginal microecology and HPV infection remains a key “Achilles heel” of HPV control programmes. Significant associations of specific vaginal microecology with HPV infection underline the value to screen for and manage aberrant vaginal microflora (i.e., those with bacteria indicative of BV) within an integrated model for HPV control among AGYW.

The major strengths of our study are the rich participant information and the utility of a culture-independent approach for the detection and/or quantification of vital organisms in the vaginal microflora. Molecular-based methods perform better than the culture method, which, for instance, cannot discriminate between *U. urealyticum* and *U. parvum*. Consequently, such culture results are inaccurately reported as *U. urealyticum* instead of ureaplasmas (56). Our findings should be considered in light of the following study limitations. In addition to its cross-sectional nature, our study included only AGYW from two schools (recruited from convenient primary care facilities) in the rural Eastern Cape, therefore may have limited its casual inferences and generalizability. Since the vaginal specimens were self-collected by inserting the brush as far as possible into the vagina after assuming a comfortable position, inter-individual differences (inconsistent sampling) in anatomical site sampled cannot be ruled out. This may have affected our results since the vaginal milieu comprises different anatomical sites, which are composed of varying microbial community composition (57). Another study limitation is the inability of the multiplex real-time PCR assay to distinguish between bacterial colonization and pathogenic infection when examining the pathobionts. We do not rule out the possibility of bias since we relied on self-reported questionnaire. Finally, we did not adjust for possible confounders for HPV infection, including sexual networks. Although not investigated, the observed HPV prevalence and its factors associated with HPV infection could somewhat be mediated by (i) poor HPV vaccine acceptance and coverage (30, 31), and (ii) poor community knowledge of HPV infection, prevention, and its consequences (31, 32, 43, 44). Notwithstanding the study limitations, our study leads us to believe that HPV education should be urgently integrated in the high school educational curriculum to confront the high HPV burden among AGYW in SSA.

## Conclusion

Our study is the first to evaluate the factors associated with HPV infection among AGYW learners in the Eastern Cape Province of South Africa. Our study, albeit not necessarily generalizable, yet consistent with published literature from other female sub-populations, found specific factors (including vaginal microecology) associated with prevalent HPV infection among unvaccinated AGYW in South Africa. The association of HPV infection and vaginal discharge or itching with specific microbes in the vaginal microflora underlines the necessity of incorporating such microbes in the surveillance and management of vaginal syndromes and infections, including HPV infection among AGYW. But first, further surveillances are needed to: (i) accurately investigate HPV epidemiology and its determinants in other AGYW populations, and (ii) adequately resolve their roles in HPV infection. To conclude, the findings from our study have the potential to inform comprehensive evidence-based interventions and programmes which, increase the public health awareness of HPV infection, its risk factors, and ramifications among AGYW in SSA.

## Data availability statement

The raw data supporting the conclusions of this article will be made available by the authors, without undue reservation.

## Ethics statement

The parent study – The HPV Education Intervention Study – was reviewed and approved by the Human Research Ethics Committee at the University of Cape Town, South Africa (HREC: 369/2015) and the Eastern Cape Province of South Africa was granted by the Eastern Cape Provincial Health Research Committee (EC_2016RP29_562). Moreover, Eastern Cape's Department of Education and Department of Health also granted us permission to conduct the study. Written informed consent to participate in parent study was provided by the participants' legal guardian/next of kin. For the present study, which sought to include a molecular test of a panel of vaginal microbes, ethical clearance was secured from the Human Research Ethics Committee at Walter Sisulu University, South Africa (protocol number: 044/2020).

## Author contributions

A-LW and ZM: conceptualization and resources. ZM: methodology, validation, supervision, project administration, and funding acquisition. HO and SM: formal analysis. HO, SM, A-LW, and ZM: investigation and writing—review and editing. HO, SM, and ZM: data curation. HO: writing—original draft preparation and visualization. All authors contributed to the article and approved the submitted version.

## Funding

The present work was funded by research grants of the South African Medical Research Council (SIR Grant Number: 384709 and SAMRC/UCT Gynaecological Cancer Research Centre) and the National Health Laboratory Service Research Trust. HO was funded by the University of Cape Town's Faculty of Health Sciences Postdoctoral Research Fellowship and the South African National Research Foundation Postdoctoral Grantholder Bursary (Grant Number: 64815).

## Conflict of interest

Author HO is employed by Tunacare Services Health Providers Limited, Nairobi, Kenya. The remaining authors declare that the research was conducted in the absence of any commercial or financial relationships that could be construed as a potential conflict of interest.

## Publisher's note

All claims expressed in this article are solely those of the authors and do not necessarily represent those of their affiliated organizations, or those of the publisher, the editors and the reviewers. Any product that may be evaluated in this article, or claim that may be made by its manufacturer, is not guaranteed or endorsed by the publisher.

## Author disclaimer

The findings, views, conclusions, and recommendations expressed in this article are those of the authors and do not necessarily represent the views of the funders and institutes affiliated with the authors and/or those mentioned in the “Acknowledgments” section above.

## Supplementary material

Supplementary Figure 1 Patterns of bacterial pathobiont infections according to vaginal HPV status among 192 AGYW. HPV-positive AGYW with **(A)** single infections, **(B)** dual infections, **(C)** triple infections; and HPV-negative AGYW with **(D)** single infections, **(E)** dual infections, **(F)** triple infections. The number of AGYW in each group is in parentheses. Of all the AGYW with information on HPV status, 193 had any detectable pathobiont (*M. genitalium, M. hominis, U. parvum*, or *U. urealyticum*). One HPV-positive participant, not included in any of the two-dimensional pie charts showing the patterns of infections, had all the four examined pathobionts detected.

## Abbreviations

β: beta
μ: micro
AGYW: adolescent girls and young women
AIDS: acquired immunodeficiency syndrome
bp: base pair
BV: bacterial vaginosis
BVAB: bacterial vaginosis-associated bacteria 2
CC: cervical cancer
CI: confidence interval
CMV: cytomegalovirus
DNA: deoxyribonucleic acid
HIV: human immunodeficiency virus
HPV: human papillomavirus
HR-HPV: high-risk human papillomavirus
HSV-1: herpes simplex virus type 1
HSV-2: herpes simplex virus type 2
IQR: interquartile range
LGV: lymphogranuloma venereum
OR: odds ratio
PCR: polymerase chain reaction
spp.: species (plural)
SSA: sub-Saharan Africa
STI: sexually transmitted infection
VZV: varicella-zoster virus
WHO: World Health Organization.
