# Supplementary figures and images for: Microbiological and behavioral determinants of genital HPV infections among adolescent girls and young women warrant the need for targeted policy interventions to reduce HPV risk
Source: Front Reprod Health. 2022 Jul 28;4:887736. doi: 10.3389/frph.2022.887736 (PMC9580722; doi:10.3389/frph.2022.887736)

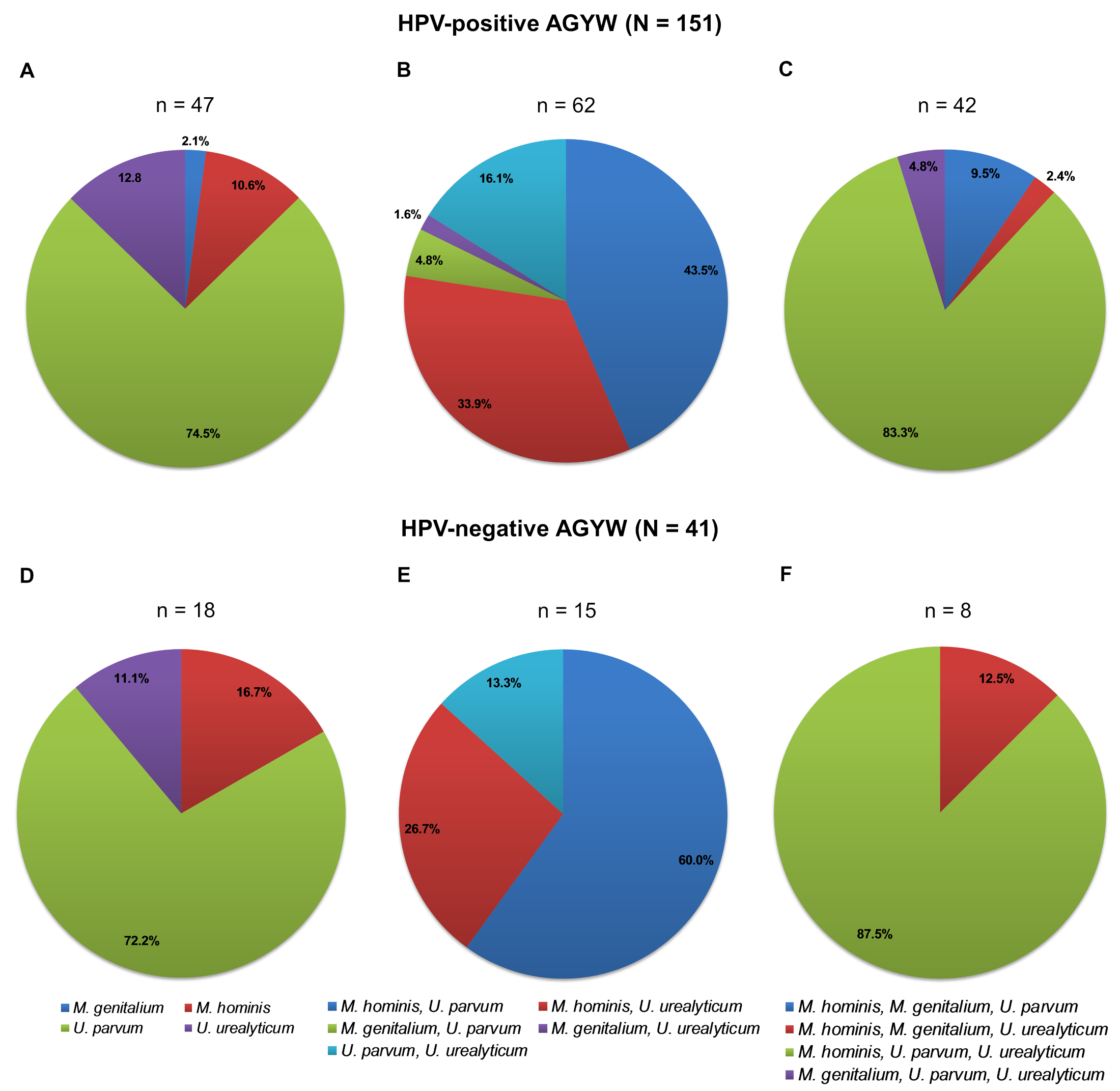

Supplement: Supplementary Figure 1 — Patterns of bacterial pathobiont infections according to vaginal HPV status among 192 AGYW. HPV-positive AGYW with (A) single infections, (B) dual infections, (C) triple infections; and HPV-negative AGYW with (D) single infections, (E) dual infections, (F) triple infections. The number of AGYW in each group is in parentheses. Of all the AGYW with information on HPV status, 193 had any detectable pathobiont (M. genitalium, M. hominis, U. parvum, or U. urealyticum). One HPV-positive participant, not included in any of the two-dimensional pie charts showing the patterns of infections, had all the four examined pathobionts detected. [file Image_1.TIF]
